# Uev1A promotes breast cancer cell survival and chemoresistance through the AKT-FOXO1-BIM pathway

**DOI:** 10.1186/s12935-019-1050-4

**Published:** 2019-12-09

**Authors:** Zhaojia Wu, Tong Niu, Wei Xiao

**Affiliations:** 1 Department of Biochemistry, Microbiology and Immunology, University of Saskatchewan, Saskatoon, SK S7N 5E5 Canada; 2 College of Life Sciences, Capital Normal University, Beijing, 100048 China

**Keywords:** Uev1A, AKT/FOXO1/BIM pathway, Cell survival, Chemoresistance

## Abstract

**Background:**

Ubiquitin-conjugating enzyme variant *UEV1A* is required for Ubc13-catalyzed K63-linked poly-ubiquitination that regulates several signaling pathways including NF-κB, MAPK and PI3K/AKT. Previous reports implicate *UEV1A* as a potential proto-oncogene and have shown that *UEV1A* promotes breast cancer metastasis through constitutive NF-кB activation. Ubc13-Uev1A along with TARF6 can also ubiquitinate AKT but its downstream events are unclear.

**Methods:**

In this study, we experimentally manipulated *UEV1* expression in two typical breast cancer cell lines MDA-MB-231 and MCF7 under serum starvation conditions and monitored AKT activation and its downstream protein levels, as well as cellular sensitivity to chemotherapeutic agents.

**Results:**

We found that overexpression of *UEV1A* is sufficient to activate the AKT signaling pathway that in turn inhibits *FOXO1* and *BIM* expression to promote cell survival under serum starvation conditions and enhances cellular resistance to chemotherapy. Consistently, experimental depletion of Uev1 in breast cancer cells inhibits AKT signaling and promotes FOXO1 and BIM expression to reduce cell survival under serum starvation stress and enhance chemosensitivity.

**Conclusions:**

Uev1A promotes cell survival under serum starvation stress through the AKT-FOXO1-BIM axis in breast cancer cells, which unveals a potential therapeutic target in the treatment of breast cancers.

## Background

Protein ubiquitination is a fundamental post-translational modification event that regulates various biological processes [1, 2]. Ubiquitination through Lys48 (K48) linked poly-Ub chain generally targets proteins for degradation by 26S proteasomes, whereas Lys63 (K63)-linked poly-Ub chain plays a critical role in various cellular events including signaling and protein trafficking [1–3]. So far, Ubc13 is the only Ub-conjugating enzyme (Ubc or E2) known to mediate the K63-linked poly-Ub chains assembly, and this process requires Uev, a Ubc/E2 variant, as a cofactor [4–7]. Uev1A and Mms2 are two major Uevs in mammalian cells. Although sharing a similar biochemical activity, they appear to function differently: Ubc13-Mms2 is required for DNA-damage response, whereas Ubc13-Uev1A is involved in NF-κB activation [7]. The Ubc13-Uev1A complex acts as an E2 and works along with Ub ligases (E3s) like TRAF6 [8, 9] and TRAF2 [10] to polyubiquitinate NEMO/IKKγ [11, 12] and/or RIP1 [13] to activate IKK. The activated IKK leads to the phosphorylation and degradation of IκBα, resulting in translocation of the NF-κB RelA (p65) subunit into the nucleus [14]. Another role of Ubc13 is in the activation of MAPK signaling [15, 16]. Ubc13 is required for the activation of mitogen-activated protein MEKK1 and TAK1, and downstream MAPK cascades on CD40 engagement in B cells [16] and Ubc13 controls breast cancer metastasis through a TAK1-p38 MAP kinase cascade [17]. An AKT kinase also undergoes Ubc13-Uev mediated K63-linked ubiquitination triggered by TRAF6, which is required for Akt membrane recruitment and subsequent phosphorylation and activation [18, 19].

*UEV1* (*CROC1* or *CIR1*) encodes at least three splicing variants, among which Uev1A and Uev1C are able to form a heterodimer with Ubc13 and promote K63-linked polyubiquitination but differ in that Uev1A contains thirty additional amino acids at the N-terminus [7, 20]. *UEV1* maps to chromosome 20q13.2 [21], a region where DNA amplification is frequently reported in breast cancers [22–24] and other tumors [25], as well as when SV40-transformed human embryonic kidney cells become immortal [26]. Moreover, *UEV1* is up-regulated in most tumor cell lines examined [20, 26, 27]. Ubc13-Uev1A involves in NF-κB activation and inhibits stress-induced apoptosis in HepG2 cells [28]. Overexpression of *UEV1A* in breast and colon cancer cells is sufficient to induce metastasis both in vitro and in vivo; this function requires Ubc13 and is mediated by NF-κB activation [20, 29]. Furthermore, a small-molecule inhibitor of Ubc13-Uev interaction can inhibit proliferation and survival of diffuse large B cell lymphoma cells [30]. These results collectively establish a positive correlation between *UEV1A* expression and tumorigenesis and metastasis.

The PI3K/Akt pathway plays an essential role in various biological functions including cell survival, proliferation, resistance to apoptosis, metabolism, differentiation, angiogenesis and migration. This pathway is frequently over-activated in human cancers and causes development of drug resistance largely due to its mediated survival signals and inhibition of apoptosis [31, 32]. It has been shown that inhibition of the PI3K/AKT pathway has a greater effect than inhibition of the MEK/MAPK pathway in enhancing the cytotoxicity of paclitaxel, doxorubicin or 5-fluorouracil [33].

One major way by which PI3K/AKT promotes cell survival is through phosphorylated inhibition the forkhead box O (FoxOs) transcription factors, such as FoxO1 and FoxO3, leading to inactivation of multiple pro-apoptotic gene expression [34, 35], such as *BCL2* family *BAX* [36, 37] and *BIM* [34, 35, 38–40].

In this study we demonstrate that in MDA-MB-231 and MCF7 breast cancer cells, overexpression of *UEV1A* alone is sufficient to activate the AKT signaling pathway that in turn inhibits *FOXO1* and *BIM* expression to promote cell survival under serum starvation stress and to enhance resistance to chemotherapy. Meanwhile, experimental depletion of Uev1 in these cells inhibits AKT signaling and increases *FOXO1* and *BIM* expression to reduce cell survival under serum starvation stress and to enhance chemosensitivity. These observations suggest a potential therapeutic target in the treatment of both triple negative (TNBC) and estrogen receptor positive (ER+) breast cancers.

## Materials and methods

### Cell lines and culture

Human breast cancer cell lines MDA-MB-231 and MCF7 were obtained from the American Type Culture Collection (ATCC). The cells were cultured in Dulbecco’s minimum essential medium (DMEM) (Invitrogen) supplemented with 10% fetal bovine serum, 100 units/ml penicillin and 100 μg/ml streptomycin (Invitrogen) in a 5% CO_2_ atmosphere at 37 °C. MDA-MB-231-TR stable cell lines were created by transfecting MDA-MB-231 cell lines with pLenti6-TR-lentivirus (Invitrogen) and selecting with 10 μg/ml blasticidin (Invitrogen).

### Plasmids and pLentivirus vector preparation

The human *UEV1A* open reading frame (ORF) was amplified and cloned into a Dox-inducible Tet-ON plasmid pcDNA4.0/TO/HA(+) as described previously [20]. The mutated Ubc13-binding site (F38E) in Uev1A was designed based on a previous study with Mms2-F13E [7]. FOXO1 (MYC-DDK-tagged)-human forehead box O1 (NM_002015) plasmid (RC200477) and pCMV6-Entry vector (PS100001) were from Origene. The human *BIM* ORF was PCR-amplified as a *Kpn*I-*Xho*I fragment and cloned into plasmid pcDNA4.0/TO/HA(+). The modified sequence for *UEV1* shRNA (sc-38606-v) and negative control shRNA (sc-108080) delivered by lentiviral particles were from Santa Cruz Biotechnology, Inc. The lentiviral particle infection of colon cancer cells was performed following instructions of the supplier.

### Survival assay and cell counting

To assess cell survival, cells were seeded in 6-well culture plates. After a 4-h exposure of cells to various doses of chemotherapeutic agents, Paclitaxel (sc-201439, Santa Cruz Biotechnology, Inc.) or Doxorubicin (sc-200923, Santa Cruz Biotechnology, Inc.), the cells were cultured for an additional 7 days with drug-free medium or medium with an PI3K/AKT pathway inhibitor LY294002 (#9901, Cell Signaling) or Perifosine (#14240, Cell Signaling), or an NF-κB pathway inhibitor Bay11-7082 (sc-200615, Santa Cruz Biotechnology, Inc.). Cells were then harvested by trypsinization at different time points and stained with trypan blue. Viable cells were counted using a hematocytometer and an inverted microscope. Each sample was measured in triplicate and repeated at least 2 times.

### RNA preparation and real-time RT-PCR (qRT-PCR)

Total RNA was prepared from MDA-MB-231 or MCF7 cells by using TRIzol reagent (Invitrogen). First-strand cDNA was synthesized from 1 μg total RNA in 20 μl reaction volume with SuperScript (Invitrogen) according to manufacturer’s instructions. qRT-PCR analysis with SYBR Green I supermix (Bio-Rad) was performed on the iQ5 cycler (Bio-Rad). The specific primer sets were as follows: *GADPH*, 5′-GAAGGTGAAGGTCGGAGTC-3′ and 5′-GAAGATGGTGATGGGATTTC-3′; *BIM*, 5′-ATGGCAAAGCAACCTTCTGA-3′ and 5′-GGATTACCTTGTGGCTCTGTCT-3′; *BCL2*, 5′-GCCCTGTGGATGACTGAGTA-3′ and 5′-CATCACCAAGTGCACCTACC-3′; *BCL6*, 5′-ATGTACCTGCAGATGGAGCA-3′ and 5′-ATCTCTGCTTCACTGGCCTT-3′; *BCL*-*xl* 5′-GGATGGCCACTTACCTGAAT-3′. and 5′-CTGCTGCATTGTTCCCATAG-3′; *FOXO1,* 5′-GGTCAAGAGCGTGCCCTACT-3′ and 5′-GCTCTTGCCACCCTCTGGAT-3′. 1 μl cDNA as template was in 20 μl reaction volume. The relative expression levels were calculated using the comparative cycle threshold (CT) method (2^−ΔCT^) [41] by the CFX Manager software (Bio-Rad).

### Western blot analysis

Total cell proteins were extracted and protein concentrations were determined as described previously [7, 20]. Cell extracts were electrophoresed in 10% or 15% SDS-PAGE gels, transferred to PVDF membrane and incubated with specific primary antibodies. The anti-Uev1 monoclonal antibody (mAb) LN2B was from the lab stock [42]. Primary antibodies against HA (sc-7392), β-tublin (sc-6216), Lamin B (sc-6216) and secondary goat anti-mouse IgG-HRP (sc- 2005) and goat anti-rabbit IgG-HRP (sc-2004) antibodies were from Santa Cruz. Primary antibodies against AKT (#4691), Phospho-Akt-Ser473 (#4060), Phospho-Akt-Thr308 (#13038), FOXO1(#2880), Phospho-FOXO1-T24 (#9464), BIM (#2933) ,PARP (#9532), IκBɑ (#4812) and Cleaved-PARP (#5941) were from Cell Signaling. Primary antibody against β-actin (TA811000s) was from Origene.

### Preparation of nuclear fraction

MDA-MB-231 and MCF7 cells were washed, scraped with PBS and centrifuged at 3000 rpm at 4 °C. The pellet was suspended in 10 mM Tris (pH 8.0) with 1.5 mM MgCl_2_, 1 mM DTT and 0.1% NP-40, and incubated on ice for 15 min. Nuclei were separated from cytosol by centrifugation at 12,000 rpm at 4 °C for 15 min. The cytosolic supernatant was removed and the precipitated pellet was suspended in 10 mM Tris (pH 8.0) containing 100 mM NaCl and stored on ice for 30 min. After agitation for 30 min at 4 °C, the lysate was centrifuged at 12,000 rpm for 15 min at 4 °C and the supernatant was collected.

### Statistical analysis

The statistical significance of differential findings between the experimental and control groups was determined by a Student’s *t*-test as implemented by Excel 2010 (Microsoft), and *P* < 0.05 was considered significant.

## Results

### *UEV1A* is involved in AKT activation and promotes cell survival under serum starvation stress

**Fig. 1:**
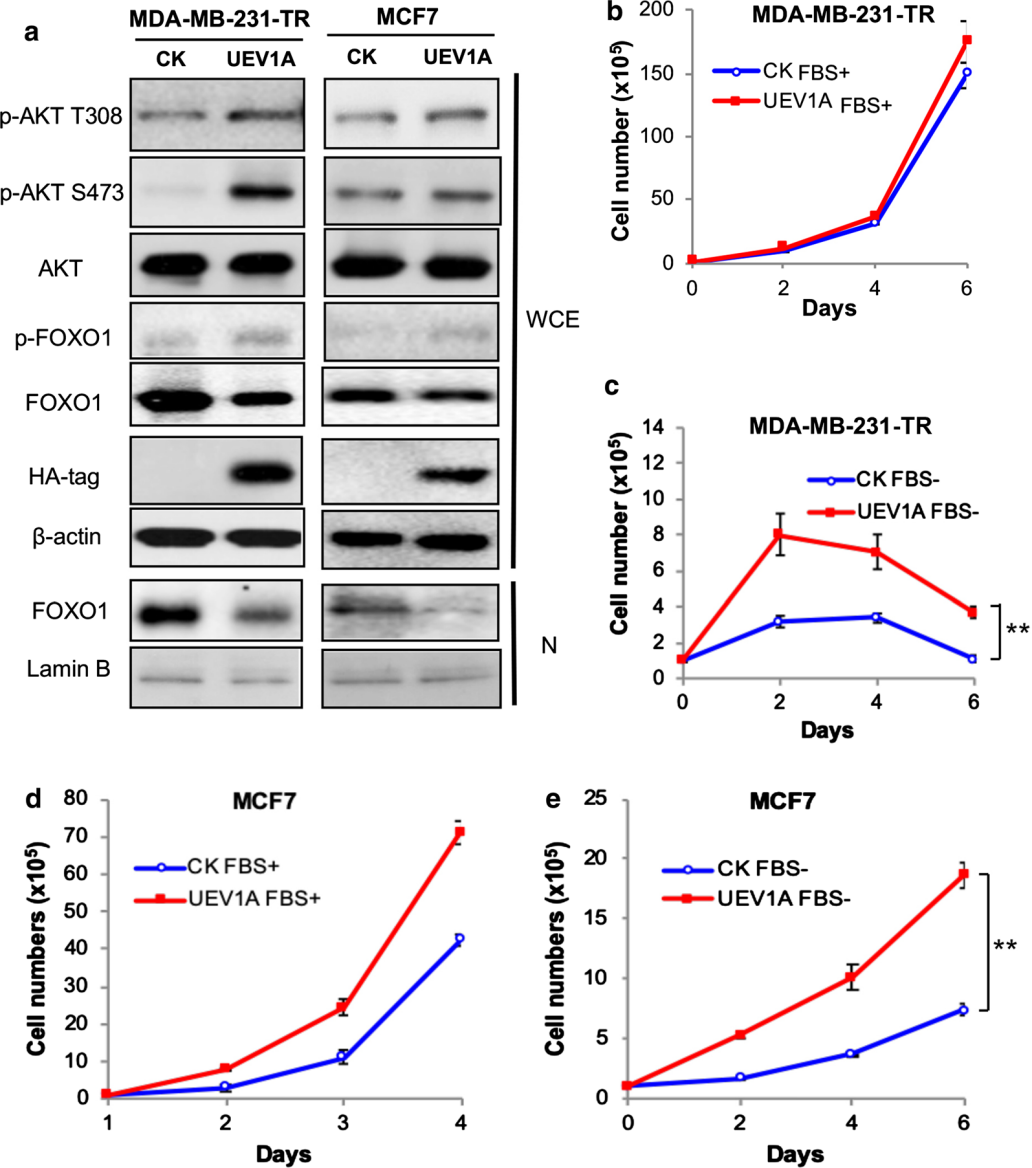
Uev1A is involved in the regulation of AKT signaling and promotes cell survival under serum starvation conditions in breast cancer cells. **a** Western blot analysis of the AKT pathway proteins and phosphorylation levels from whole-cell extract (WCE) or nuclear fraction (N) in pcDNA4.0/TO/HA(+) vector (CK) or pcDNA4-*UEV1A* stably transfected MDA-MB-231 tet-on (TR) cells treated with doxycycline (left panel) or in MCF7 cells (right panel). The ectopic Uev1A levels were monitored by an anti-HA antibody. **b**, **c** Growth curve of control (CK) and *UEV1A* overexpression MDA-MB-231-TR cells under serum-supplemented and serum-deprived conditions. Cells were treated with (**b**) or without (**c**) 10% FBS and then harvested by trypsinization at indicated time points and stained with trypan blue. Viable cells were counted using a hematocytometer and an inverted microscope. Each sample was measured in triplicate and repeated 2 times. **d**, **e** Growth curve of control (CK) and *UEV1A* overexpression MCF7 cells under serum-supplemented and serum-deprived conditions. Experimental conditions were as described in **b**, **c**. **P < 0.01

To ask whether an elevated *UEV1A* level is sufficient to regulate AKT signaling in breast cancer cells, *UEV1A*, *UEV1C* or *MMS2* were cloned into a pcDNA4.0/TO/HA(+) vector and then transfected into MDA-MB-231-TR or MCF7 cells to construct stable cell lines as previously described [20]. The level of ecotopic gene expression was monitored by western blot against the HA-tag. Then the phosphorylation levels of AKT at S473 and T308 were detected by western blot. As seen in Fig. 1a, the AKT phosphorylation increased at both S473 and T308 residues after *UEV1A* overexpressed in MDA-MB-231-TR and MCF7 cells. In contrast, overexpression of *UEV1C* or *MMS2* did not cause AKT phosphorylation at S473 or T308, but rather slightly reduced the phosphorylation at these sites (Additional file 1: Figure S1A, B). Since *UEV1A* overexpression mainly caused the AKT-S473 phosphorylation, which appears to be more critical than AKT-T308 phosphorylation for its full activation [43], we subsequently focused only on monitoring AKT-S473 phosphorylation.

It has been reported that many cultured cells undergo apoptosis when grown in serum-free culture medium [44] and our previous results showed that *UEV1A* overexpression promoted HepG2 cell growth in a serum-free medium [28]. We then examined cell growth of *UEV1A*-overexpressed MDA-MB-231-TR or MCF7 cells under serum-supplemented and serum-deprived conditions. The growth curve of *UEV1A*-overexpressed MDA-MB-231-TR cells was not significantly different from that of control cells when serum was supplemented (Fig. 1b). However, with serum deprivation, *UEV1A*-overexpressed cells grew much faster than the control cells, reaching up to 3.4-fold in cell numbers (Fig. 1c). In MCF7 cells, although *UEV1A*-overexpressed cells had a higher growth rate than control under both serum-supplemented and serum-deprived conditions, the number of *UEV1A*-overexpressed live cells was approximately 2.53-fold that of control after 6 days culturing in serum-free medium (Fig. 1e), whereas the difference was approximately 1.67-fold in the presence of serum (Fig. 1d). To further strengthen the above observations, we also performed a colony formation assay under serum-deprived conditions, and found that overexpression of *UEV1A* significantly increased number of colonies in MDA-MB-231 and MCF7 cells (Additional file 1: Figure S2).

### Endogenous Uev1A is required for the AKT signaling pathway

The above observations led us to hypothesize that *UEV1A* prevents breast cancer cell apoptosis through the AKT signaling pathway. It is well established that AKT can phosphorylate and inhibit pro-apoptotic proteins like FOXO family proteins to prevent apoptosis [34, 35]. We then examined total and phosphorylated protein levels of FOXO1 in MDA-MB-231-TR or MCF7 cells by western blot in both whole-cell extracts (WCE) and the nuclear (N) fraction. Upon *UEV1A*-overexpression, the total FOXO1 protein level was decreased while phosphorylated FOXO1 was increased (Fig. 1a). Meanwhile, the nuclear FOXO1 protein level was also decreased (Fig. 1a). These results suggest that *UEV1A*-promoted cell survival under serum starvation stress is related to the AKT/FOXO1 pathway.

**Fig. 2:**
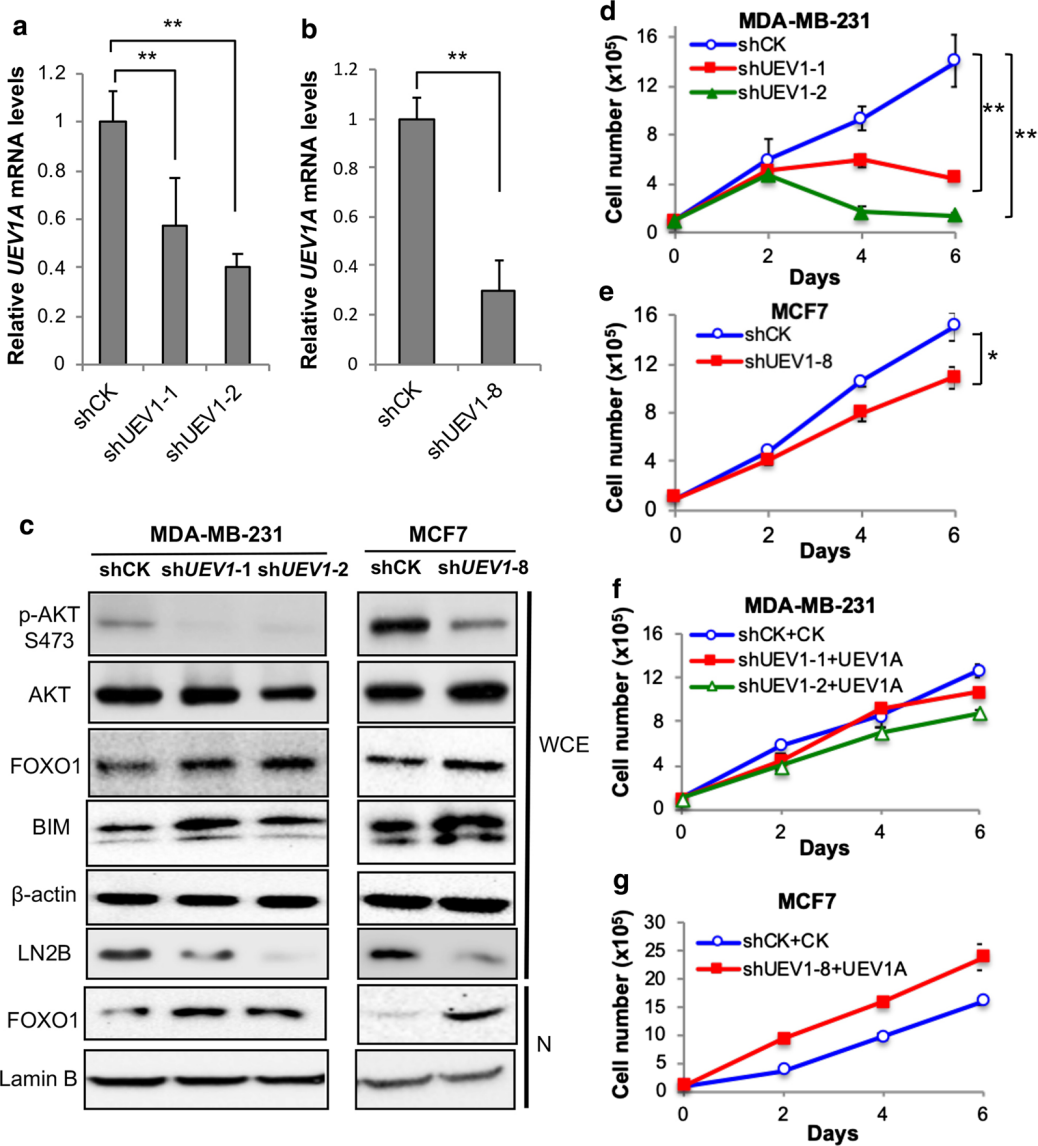
Uev1 depletion inactivates AKT pathway signaling and reduces cell survival under serum starvation conditions in breast cancer cells. MDA-MB-231 or MCF7 cells were transfected with shRNA lentiviral particles against *UEV1* (sh*UEV1*) or non-specific target (shCK). shUEV1-1 and shUEV1-2 represent two independent stable shUEV1 MDA-MB-231 cell lines. shUEV1-8 represents a stable shUEV1 MCF7 cell line. **a** *UEV1A* transcript levels in shCK and shUEV1 lines were determined by qRT-PCR in MDA-MB-231 cells. **b** *UEV1A* transcript levels in shCK and shUEV1 line were determined by qRT-PCR in MCF7 cells. **c** AKT pathway proteins in whole-cell extracts (WCE) or nuclear fractions (N) were detected by western blot. The endogenous Uev1 was monitored by LN2B antibody. **d** Growth curve of non-specific target (shCK) and shUEV1 MDA-MB-231 cell lines (shUEV1-1 and shUEV1-2) under serum-deprived conditions. Then cells were harvested and cell numbers were determined as described in Fig. 1b, c. **e** Growth curve of non-specific target (shCK) and shUEV1 MCF7 cell line (shUEV1-8) under serum-deprived conditions. Then cells were harvested and cell numbers were determined as described in Fig. 1b, c. **f** Two shUEV1 MDA-MB-231 cell lines were transfected with the pcDNA4.0/TO/HA(+) vector expressing *UEV1A* (shUEV1-1 + UEV1A and shUEV1-2 + UEV1A). Non-specific shRNA targeted MDA-MB-231 cells were also transfected with the pcDNA4.0/TO/HA(+) vector to serve as a control. Cells cultured under serum-deprived conditions were harvested and cell numbers were determined as described in Fig. 1b, c. **g** The MCF7 shUEV1-8 cell line was transfected with pcDNA4.0/TO/HA(+) vector expressing *UEV1A* (shUEV1-8 + UEV1A). The non-specific shRNA targeted MCF7 cells were transfected with the vector alone (shCK + CK). Cells cultured under serum-deprived conditions were harvested and cell numbers were determined as described in Fig. 1b, c. Each sample was measured in triplicate and repeated at least 2 times. *P < 0.05; **P < 0.01

It has been previously reported that the *UEV1A* level may be elevated when normal cells undergo immortalization [39] and *UEV1A* transcript level is elevated in all breast cancer cell lines examined [20]. To ask whether the moderate elevation of *UEV1A* levels in breast cancer cells contributes to the AKT pathway activation, the endogenous *UEV1A* expression in MDA-MB-231 and MCF7 cells was suppressed using an shRNA (shUEV1) delivered by lentiviral particles. The independent shUEV1 cell lines were constructed as previously described [20, 29]. The *UEV1A* mRNA levels of shUEV1 cell lines were shown in Fig. 2a, b. The endogenous protein level of Uev1 was detected by western blot using an anti-Uev1 mAb LN2B [42]. Depletion of Uev1 dramatically reduced the AKT-S473 phosphorylation and increased its downstream FOXO1 and BIM protein levels (Fig. 2c). Furthermore, depletion of Uev1 decreased cell growth under serum deprivation condition (Fig. 2d, e) in comparison to that under serum-supplemented conditions (Additional file 1: Figure S3A, B). Importantly, *UEV1A* overexpression in Uev1-depletion cells (Additional file 1: Figure S4A, B) can reverse the retarded growth caused by Uev1 depletion under serum deprivation conditions (Fig. 2f, g), which supports a notion that the AKT pathway is regulated by Uev1A instead of Uev1C.

### *UEV1A* promotes cell growth through AKT/FOXO1 but not the NF-κB pathway

**Fig. 3:**
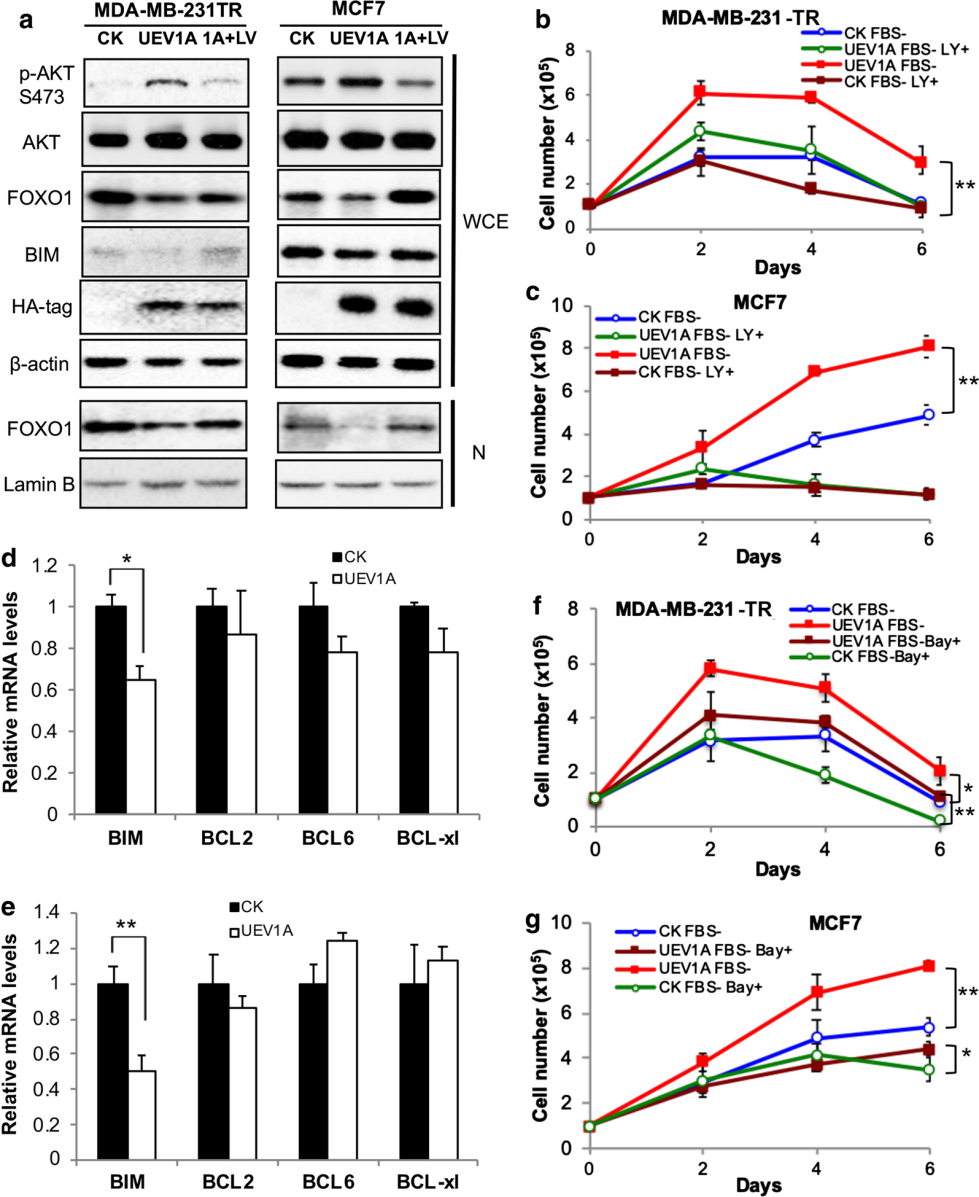
Uev1A promotes cell survival under serum starvation conditions through the AKT but not NF-κB pathway in breast cancer cells. **a** *UEV1A* overexpressed MDA-MB-231-TR cells (left panel) and MCF7 cells (right panel) were treated with LY294002. After 24 h, the AKT pathway proteins were examined by western blot in the whole-cell extract (WCE) or nuclear fraction (N) in *UEV1A* overexpressed cells alone (UEV1A) or treated with 10 μM LY294002 (1A + LY), or vector only (CK). The expression levels of ectopic Uev1A were monitored by an anti-HA antibody. **b**, **c** Growth curve of control (CK) and *UEV1A*-overexpressed MDA-MB-231-TR (**b**) and MCF7 (**c**) cells under serum-deprived conditions. Experimental conditions were as described in Fig. 2c except that some cells were treated with 10 μM LY294002 (LY+). **d**, **e** Effects of *UEV1A* overexpression on the expression of selected FOXO1 target genes. Transcript levels of putative FOXO1 target genes in MDA-MB-231-TR (**d**) or MCF7 (**e**) cells overexpressing *UEV1A* as determined by qRT-PCR. **f**, **g** Growth curve of control (CK) and *UEV1A*-overexpressed MDA-MB-231-TR (**f**) and MCF7 (**g**) cells under serum-deprived conditions. Experimental conditions were as described in Fig. 2c except that some cells were treated with 40 μM Bay11-7082 (Bay+). Each sample was measured in triplicate and repeated at least 2 times. *P < 0.05; **P < 0.01

To ask whether Uev1A promotes cell survival in serum-deprived conditions through the AKT/FOXO1 pathway, we examined effects of two PI3K/AKT pathway inhibitors LY294002 [45] and Perifosine [46] on *UEV1A* overexpressed MDA-MB-231-TR or MCF7 cells. The phosphorylation level of AKT at S473 and FOXO1 protein level were detected by western blot to confirm the blocking of AKT activation after LY294002 or Perifosine treatment. Compared to cells without the inhibitor treatment, the phosphorylation level of AKT-S473 was clearly reduced after LY294002 (Fig. 3a) or Perifosine (Additional file 1: Figure S5A) treatment. Meanwhile total and nuclear FOXO1 levels were increased in both cell lines (Fig. 3a and Additional file 1: Figure S5A). We then examined cell growth under serum-deprived conditions and found that, compared to cells without the PI3K/AKT pathway inhibitor treatment, the growth of *UEV1A*-overexpressed MDA-MB-231-TR cells was reduced after LY294002 or Perifosine treatment to a level comparable to control cells (Fig. 3b and Additional file 1: Figure S5B). Similarly, compared to cells without the inhibitor treatment, the growth of *UEV1A*-overexpressed MCF7 cells was also reduced after LY294002 or Perifosine treatment to the control cell level (Fig. 3c and Additional file 1: Figure S5C). As FOXO1 is a member of the forkhead-box transcription factors and it regulates genes involved in apoptosis [34], we measured the mRNA level of FOXO1 downstream genes by qRT-PCR. The BIM mRNA level was significantly decreased in *UEV1A*-overexpressed MDA-MB-231-TR (Fig. 3d) and MCF7 (Fig. 3e) cells. Similarly, the BIM protein level was decreased in *UEV1A*-overexpressed MDA-MB-231-TR and MCF7 cells and was increased after LY294002 treatment (Fig. 3a). These results indicate that Uev1A promotes cell survival under serum starvation stress through the AKT/FOXO1/BIM pathway. Since Uev1A has also been reported to be involved in NF-κB activation [20, 28, 29], we asked if Uev1A also promotes cell survival through the NF-κB pathway. An NF-κB inhibitor Bay11-7082 [47] was used to treat cells and its efficicacy was validated by monitoring the nuclear p65 level (Additional file 1: Figure S6). As the growth of *UEV1A*-overexpressed MDA-MB-231-TR (Fig. 3f) or MCF7 (Fig. 3g) cells under serum-deprived conditions was still significantly different from that of vector control cells after treatment with Bay11-7082, indicating that Uev1A promoted breast cancer cell survival under serum starvation stress is independent of the NF-κB pathway.

### BIM is the downstream effactor for Uev1A-promoted cell survival under serum starvation stress

**Fig. 4:**
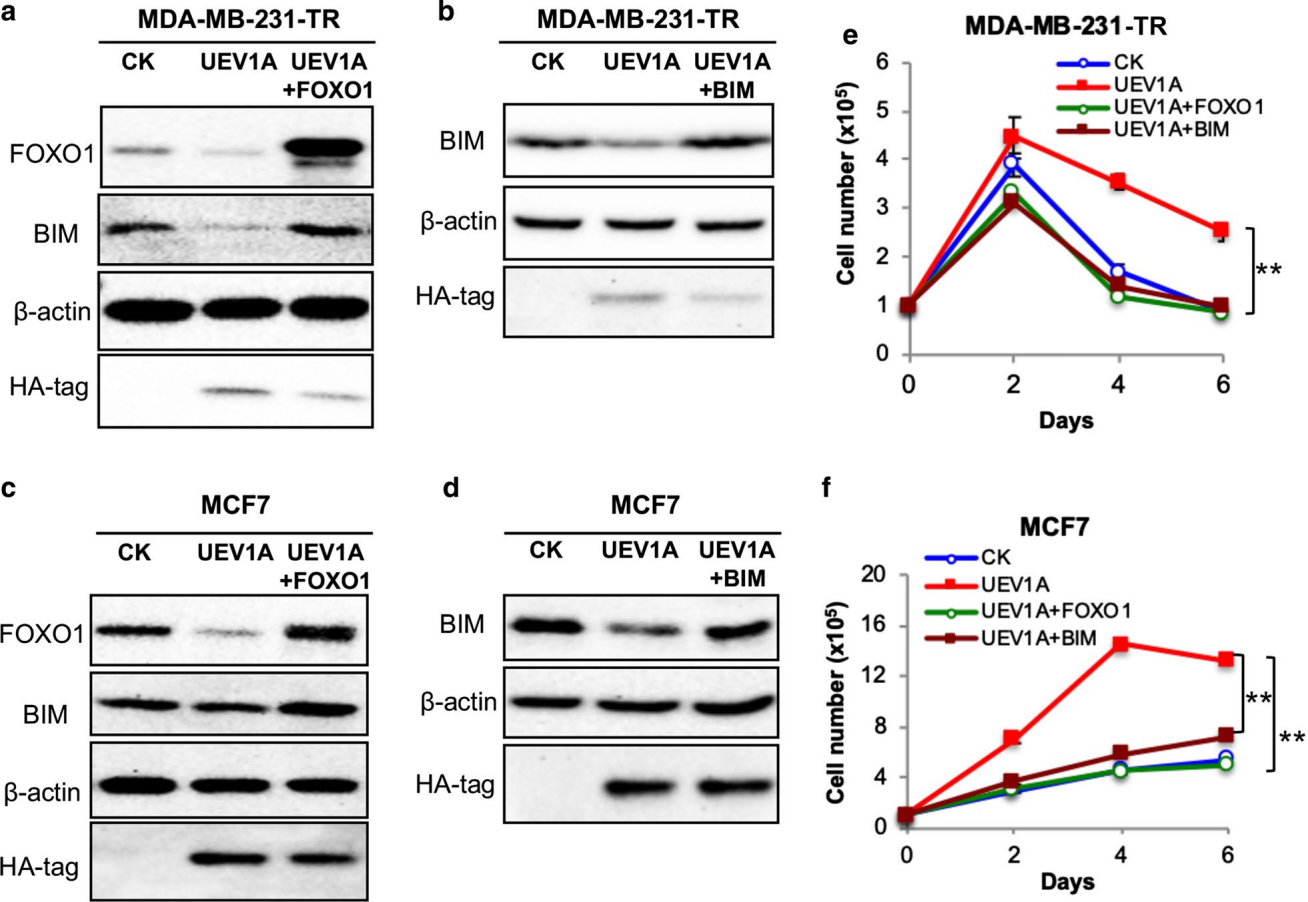
BIM is a downstream effector for Uev1A to promote cell survival under serum starvation conditions in breast cancer cells. **a**, **c** pCMV6-*FOXO1* was transfected into *UEV1A*-overexpressed MDA-MB-231-TR (**a**) or NCF7 (**b**) cells. The protein levels of ectopic FOXO1 and BIM were detected by western blot. The protein levels of ectopic Uev1A were monitored by an anti-HA antibody. **b**, **d** pcDNA4.0/TO/HA(+)-*BIM* was transfected into *UEV1A*-overexpressed MDA-MB-231-TR (**b**) or MCF7 (**d**) cells. The protein levels of ectopic BIM were monitored by western blot. Ecotopic Uev1A levels were monitored by an anti-HA antibody. **e**, **f** Growth curve of four groups of MDA-MB-231-TR (**e**) or MCF7 (**f**) cells under serum-deprived conditions. Vector only (CK), *UEV1A*-overexpressed (UEV1A), *FOXO1*- and *UEV1A*-overexpressed (UEV1A + FOXO1) and *BIM*- and *UEV1A*-overexpressed (UEV1A + BIM). All MDA-MB-231-TR cells were cultured with doxycycline. Each sample was measured in triplicate and repeated at least 2 times. **P < 0.01

To ask if BIM is a critical effector for Uev1A-induced cell survival under serum starvation stress, we overexpressed *FOXO1* or *BIM* in *UEV1A*-overexpressed cells to restore *FOXO1* or *BIM* protein levels. The ecotopic expression was assessed by western blot in MDA-MB-231-TR (Fig. 4a, b) and MCF7 (Fig. 4c, d) cells. As expected, in both MDA-MB-231-TR (Fig. 4a) and MCF7 (Fig. 4c) cells, the BIM protein level was elevated in *FOXO1*-overexpressed cells. Then we examined cell growth under serum-deprived conditions and found that in both MDA-MB-231-TR (Fig. 4e) and MCF7 (Fig. 4f) cells, overexpression of either *FOXO1* or *BIM* in *UEV1A*-overexpressed cells significantly decreased cell growth to the level comparable to that of vector control. The above findings allow us to conclude that Uev1A promotes cell survival under serum starvation stress via the AKT-FOXO1-BIM axis.

### Uev1A-activated AKT pathway is dependent on Ubc13

**Fig. 5:**
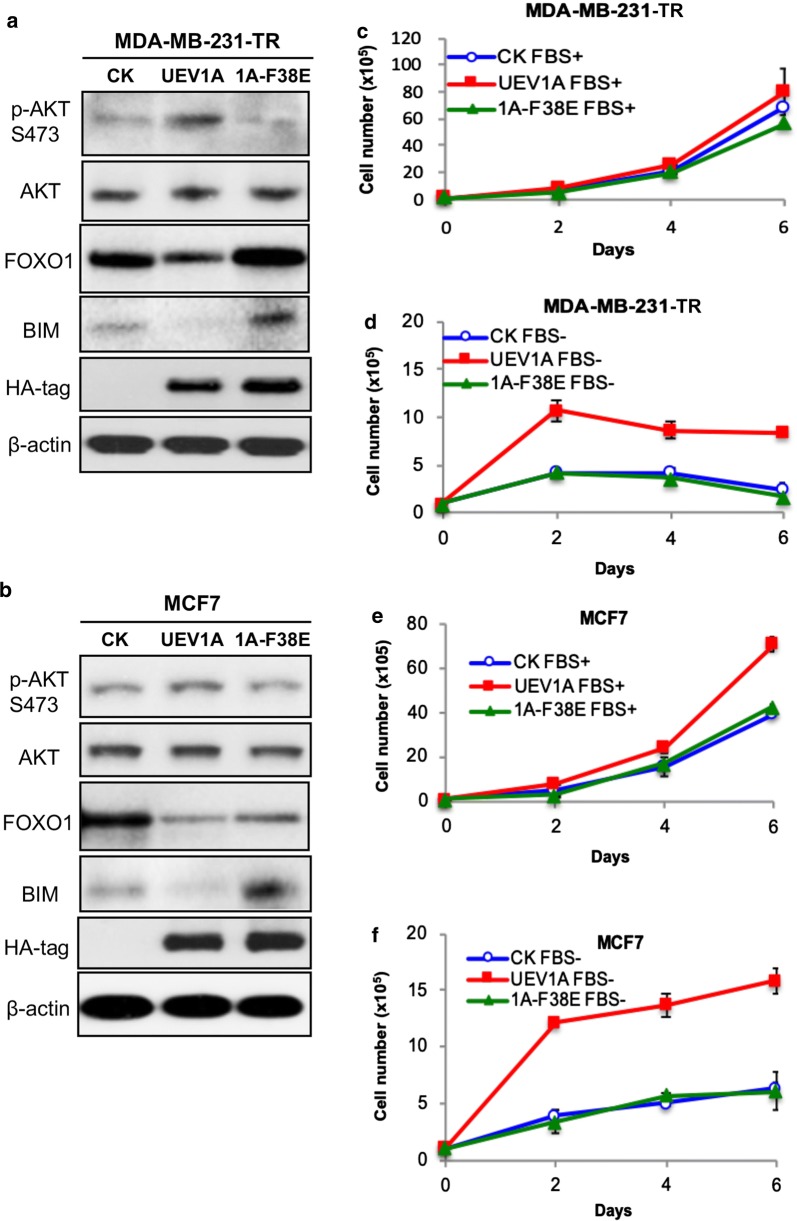
Uev1A promotes cell survival under serum starvation conditions through AKT pathway in a Ubc13-dependent manner. **a**, **b** The protein levels and phosphorylation levels of AKT pathway proteins were detected by western blot in pcDNA4.0/TO/HA(+) vector expressing *UEV1A, UEV1A*-*F38E* (1A-F38E) or vector only (CK) stably transfected MDA-MB-231-TR cells treated with doxycycline (**a**) or MCF7 (**b**) cells. **c**, **d** Growth curve of control (CK) and *UEV1A*-*F38E*-overexpressed (1A-F38E) MDA-MB-231-TR cells under serum-supplemented (**b**) and serum-deprived (**c**) conditions. **e**, **f** Growth curve of control (CK) and *UEV1A*-*F38E*-overexpressed (1A-F38E) MCF7 cells under serum-supplemented (**e**) and serum-deprived (**f**) conditions. Each sample was measured in triplicate and repeated at least 2 times

It has been previously reported that Uev1A is a cofactor of Ubc13 to trigger K63-linked polyubquitination [5, 6, 48]. To ask whether the above Uev1A function is indeed dependent on Ubc13, we created a Uev1A-F38E mutation that abolishes its interaction with Ubc13 and ability to promote Ubc13-mediated K63-linked polyubiquitination [7]. As expected, overexpression of *UEV1A*-*F38E* in MDA-MB-231TR (Fig. 5a) and MCF7 (Fig. 5b) cells failed to activate the AKT pathway, as judged by reduced AKT-S473 phosphorylation and increased FOXO1 and BIM. Correspondingly, overexpression of *UEV1A*-*F38E* did not increase MDA-MB-231-TR (Fig. 5c, d) and MCF7 (Fig. 5e, f) cell growth either with serum-supplemented or serum deprived conditions. Taken together, we conclude that *UEV1A* overexpression facilitates the Uev1A-Ubc13 complex formation, which promotes AKT signaling and cell survival under serum starvation stress.

### Uev1A promotes AKT-mediated chemoresistance in breast cancer cells

**Fig. 6:**
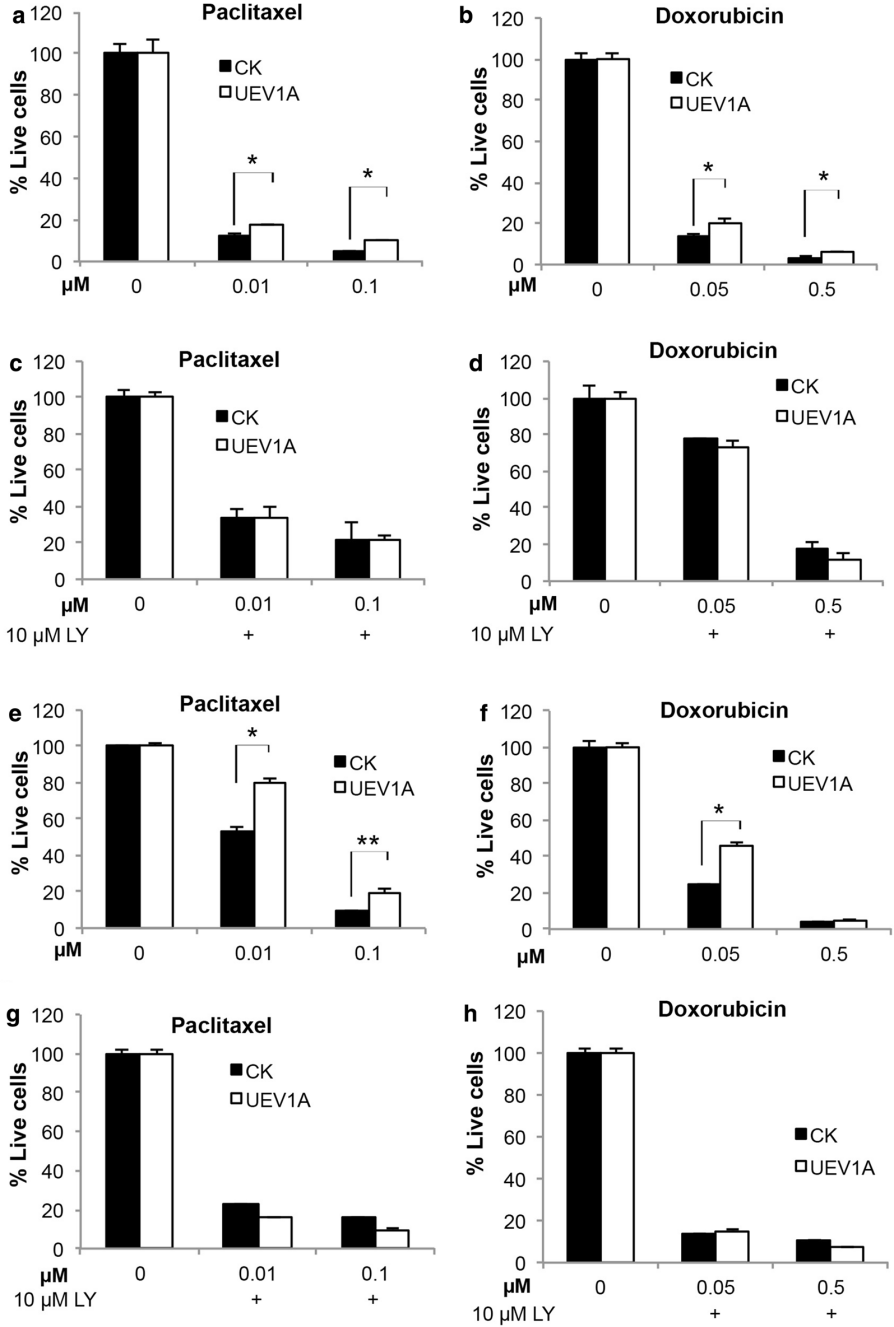
Uev1A promotes AKT-mediated chemoresistance in breast cancer cells. **a**, **b** Effects of *UEV1* overexpression on chemoresistance of MDA-MB-231 cells. *UEV1A*-overexpressed or vector control MDA-MB-231-TR cells were seeded onto 6-well culture plates with doxycycline. After a 4-h exposure to various doses of chemotherapeutic agents Paclitaxel (**a**) or Doxorubicin (**b**), the cells were cultured for an additional 7 days with drug-free medium containing 10% FBS. Then cells were harvested by trypsinization and stained with trypan blue. Cell viability was assayed by cell number counting using a hematocytometer and an inverted microscope. Each sample was measured in triplicate and repeated 2 times. **c**, **d** Dependence of chemoresistance of MDA-MB-231 cells to the AKT pathway. *UEV1A*-overexpressed or vector control MDA-MB-231-TR cells were seeded onto 6-well culture plates with doxycycline. With 10 μM LY294002 pretreated for 12 h, cells were exposed to various doses of Paclitaxel (**c**) or Doxorubicin (**d**) which were also in the medium with 10 µM LY294002 for 4 h and then cultured for an additional 7 days with medium containing 10% FBS and 10 µM LY294002. Cell viability assay was as described in **a**, **b**. **e**, **f** Effects of *UEV1* overexpression on chemoresistance of MCF7 cells. Experimental conditions were as described in **a**, **b**. **g**, **h** Dependence of chemoresistance of MCF7 cells to the AKT pathway. Experimental conditions were as described in **c**, **d**. *P < 0.05; **P < 0.01

Chemotherapy is the only choice of treatment for triple negative cancer (TNBC), and treatment of choice for estrogen receptor positive (ER+) breast cancer. However, chemoresistance is still a major limitation in breast cancer treatment. It has been reported that AKT pathway activation is involved in the resistance to many chemotherapy drugs. To ask weather Uev1A contributes to chemoresistance of breast cancer cells, we used two representative chemotherapeutic drugs, Paclitaxel and Doxorubicin, to treat *UEV1A*-overexpressed MDA-MB-231-TR cells and MCF7 cells. After 4-h exposure to various doses of chemotherapeutic drugs, the cells were cultured for an additional 7 days with drug-free medium containing 10% FBS followed by assessment of cell survival. Indeed, overexpression of *UEV1A* in MDA-MB-231-TR cells significantly increased the number of live cells treated with Paclitaxel (Fig. 6a) or Doxorubicin (Fig. 6b) compared with vector control cells, and this difference was abolished by adding LY294002 to the chemotherapeutic drugs treatment (Fig. 6c, d). Furthermore, significant decrease in MDA-MB-231 cell survival was observed after Paclitaxel and Doxorubicin treatment when endogenous Uev1 was depleted (Additional file 1: Figure S7A, B). Similar phenomena were also observed in MCF7 cells (Fig. 6e–h, and Additional file 1: Figure S7C, D). These results indicate that Uev1A promotes chemoresistance through the AKT pathway in both in triple negative and estrogen receptor positive (ER+) breast cancers.

### Uev1A inhibits apoptosis through the AKT pathway in breast cancer cells

**Fig. 7:**
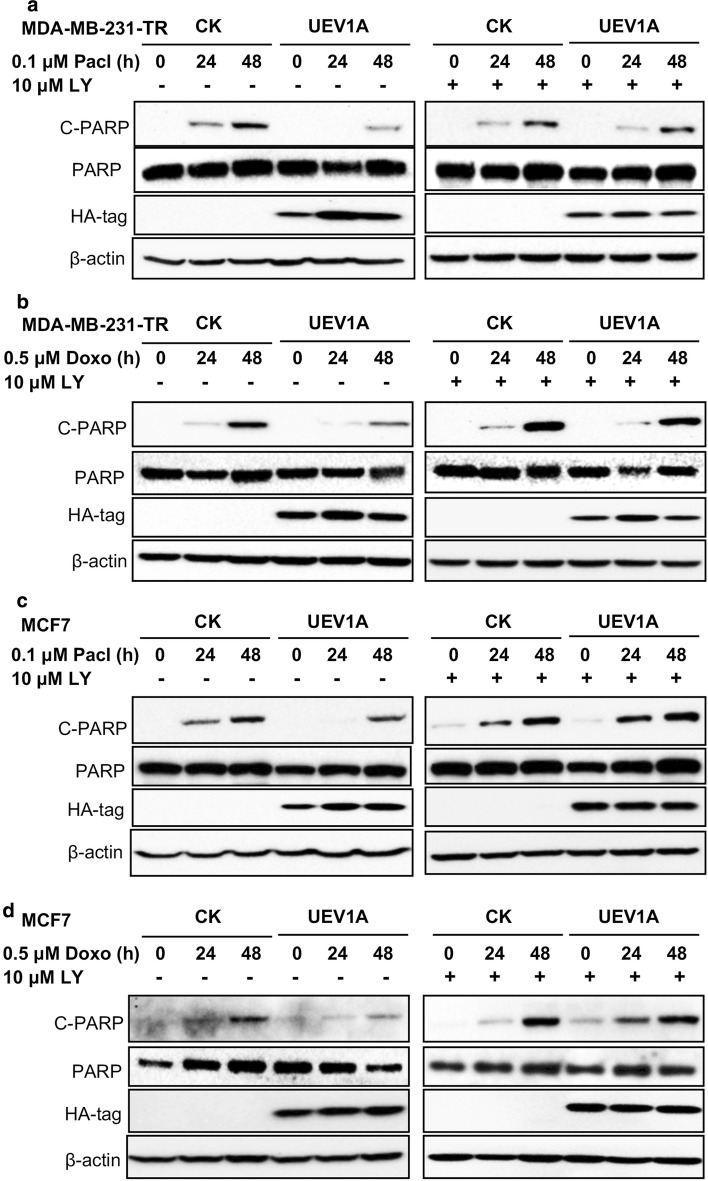
Uev1A inhibits apoptosis through the AKT pathway in breast cancer cells. **a**, **b** *UEV1A*-overexpressed or vector control MDA-MB-231-TR cells with Paclitaxel (Pacl) (**a**) or Doxorubicin (Doxo) (**b**). **c**, **d** *UEV1A*-overexpressed or vector control MCF7 cells with Paclitaxel (Pacl) (**c**) or Doxorubicin (Doxo) (**d**). Cells were pretreated with (right panel) or without (left panel) 10 µM LY294002 (LY) for 12 h and then exposed to Paclitaxel or Doxorubicin, harvested at different time points and the protein levels of total PARP and cleaved-PARP (C-PARP) were detected by western blot. The ectopic Uev1A was monitored by an anti-HA antibody

To further confirm that Uev1A inhibits apoptosis through the AKT pathway, we examined the protein level of cleaved-PARP, a well-established apoptosis marker [49, 50], in MDA-MB-231-TR and MCF7 cells after Paclitaxel or Doxorubicin treatment. Cells were exposed to Paclitaxel or Doxorubicin for 24 and 48 h, and cleaved-PARP was detected by western blot. Compared with vector control, overexpression of *UEV1A* significantly delayed the appearance of cleaved-PAPR in both MDA-MB-231TR (Fig. 7a, b) and MCF7 (Fig. 7c, d) cells after Paclitaxel (Fig. 7a, c) or Doxorubicin (Fig. 7b, d) treatment. Furthermore, the difference between *UEV1A*-overexpressed cells and vector control cells were abolished when LY294002 or Perifosine was added along with the chemotherapeutic drug treatment (Fig. 7a–d and Additional file 1: Figure S8A–D). Hence, we conclude that Uev1A inhibits apoptosis through the AKT pathway in breast cancer cells.

## Discussion

AKT signaling is essential for many biological processes such as cell proliferation and apoptosis. It has been clear that AKT kinase undergoes K63-linked ubiquitination triggered by TRAF6, which is required for AKT membrane recruitment and subsequent AKT phosphorylation and activation [18, 19]. However, no study has focused on the involvement of a key factor, the Ubc13-Uev1A complex, during AKT activation. As the only known E2 to mediate K63-linked poly-Ub chains assembly, Ubc13-Uev1A likely mediates AKT ubiquitination, but the downstream events of Ubc13-Uev1A in the AKT pathway remains unclear. This study demonstrates that overexpression of *UEV1A* alone is sufficient to activate AKT, which promotes cell survival and chemoresistance in breast cancer cells. Since the cellular Ubc13 level is considered abundant in human cells, Uev1A is likely a limiting factor in the regulation of AKT pathway.

Human cells contain two *UEV* genes, *UEV1* and *MMS2* whose products share > 90% amino acid sequence identity in their core domains [27]. Although both Uev1A and Mms2 are cofactors of Ubc13 that together mediate K63-linked polyubiquitination, their biological functions are apparently distinct and only the Uev1A-Ubc13 complex is involved in NF-κB signaling and promotes metastasis in breast and colon cancers [7, 20, 28, 29]. Furthermore, there are at least three splicing variants of *UEV1*, among which Uev1C are also able to bind Ubc13 and promote K63-linked poly-ubiquitination as Uev1A. However, Uev1C does not contribute to NF-κB activation and are not able to promote metastasis in breast and colon cancers [20, 29]. This study investigated roles of *UEV1A*, *UEV1C* and *MMS2* in AKT pathway activation in two breast cancer cell lines. Not surprisingly, with comparable levels of ectopic expression, only *UEV1A*, but not *UEV1C* or *MMS2*, is able to promote AKT pathway activation. In a reverse experiment, depletion of Uev1 in two breast cancer cell lines significantly inhibits AKT pathway activation, indicating that the cellular Uev1 (presumbly Uev1A) level plays a critical role in oncogenic AKT activation. Although the exact function of Uev1C remains unclear, lack of the N-terminal domain found in Uev1A determines its subcellular localization and involvement in different pathways [7, 20].

**Fig. 8:**
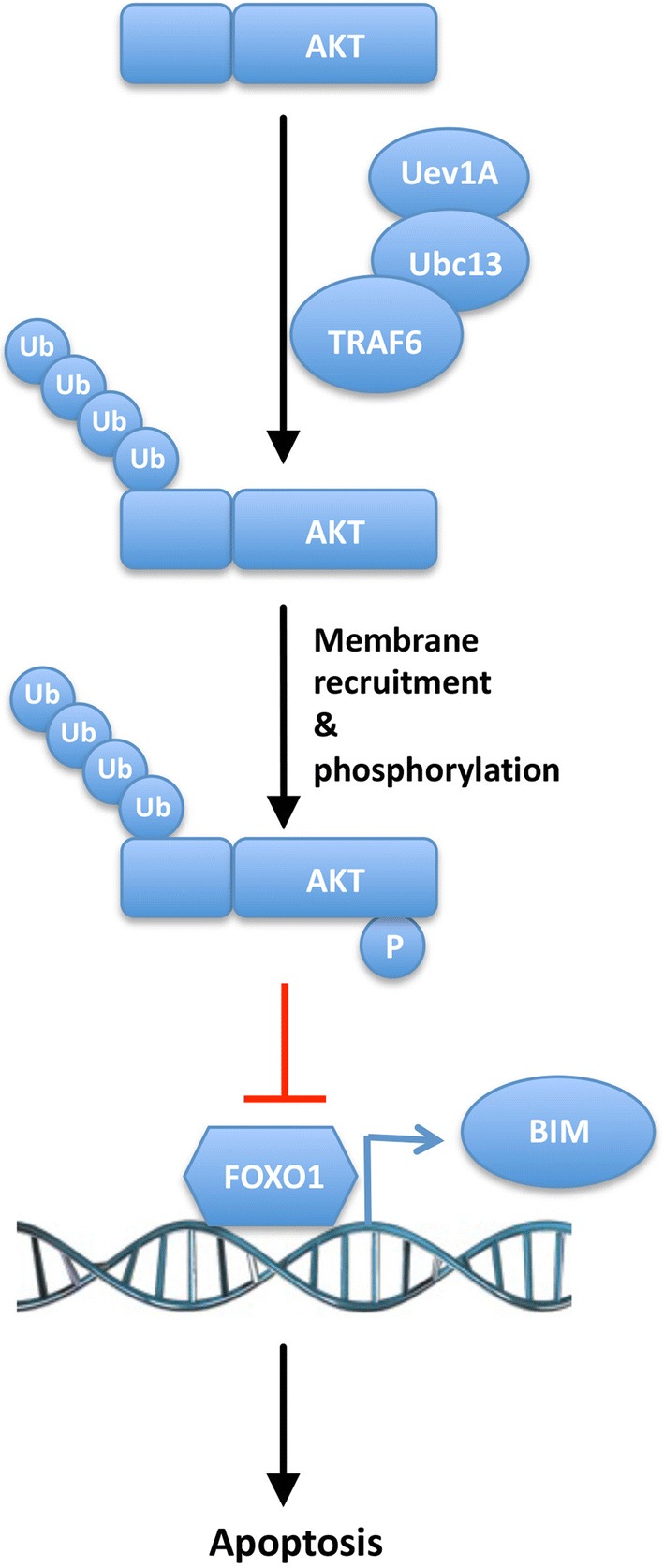
Uev1A promotes cell survival through the AKT-FOXO1-BIM axis in breast cancer cells. The Ubc13-Uev1A complex works as an E2 along with TRAF6 to ubiquitinate AKT, which is required for the AKT membrane recruitment and subsequent phosphorylation and activation. The AKT phosphorylation negatively regulates the transcription factor FOXO1 that induces the expression of a pro-apoptosis gene *BIM* to trigger apoptosis

Among FOXO members, the FOXO1 activity is negatively regulated by PI3K/AKT, which phosphorylates FoxO1 at multiple sites and forces FoxO1 degradation and translocation into the cytoplasm [51]. The FOXO1 transcription factors are important regulators of cell cycle arrest and apoptosis, functioning as tumor suppressors [34, 35, 52]. Under stress conditions, FOXO1 induces expression of a pro-apoptosis gene *BIM* to trigger programmed death of overstressed/damaged cells [39, 40, 53]. Furthermore, overexpression of *FOXO1* or *BIM* can significantly decrease cell growth in *UEV1A*-overexpressed cells under serum-deprived conditions. Meanwhile, Bim is a major mediator of anticancer drug-induced cell death [38, 54]. Therefore, induction of *BIM* by FOXO1 may be a key step in maintaining the BIM level in breast cancer cells and dictating their response to chemotherapy drugs. Our data collectively suggest that Uev1A promotes cell survival under serum starvation stress and drugs resistance through the AKT-FOXO1-BIM axis in breast cancer cells, as outlined in Fig. 8.

It has been more than 20 years since *UEV1* (*UEV1A*) was proposed as a proto-oncogene. Although *UEV1A* is abnormally overproduced in a variety of tumors and it is involved in NF-κB activation and tumor metastasis, our understanding of the Uev1A function is just the tip of the iceberg. This study reveals that Uev1A can activate AKT, promote cell survival and enhance chemoresistance in breast cancer cells. In fact, it has been reported that K63-linked ubiquitination triggered by TRAF6 regulates multiple signal transduction pathways, such as NF-κB, MAPKs and AKT, all of which relate to tumorigenesis. As the only known Ubc/E2 that works with TRAF6 and promotes K63-linked polyubiquitination, the Ubc13-Uev1A complex should also be involved in the above pathways. In addition, AKT is ubiquitinated at its PH domain [55, 56], which is also found in other protein kinases [57, 58], and, like AKT, these kinases also contain the TRAF6 consensus binding motif, raising a possibility that TRAF6-Ubc13-Uev1A induces ubiquitination of these kinases [19]. Research on potential functions of *UEV1A* may further advance our understanding of how *UEV1A* works as a proto-oncogene.

## Conclusion

Our studies suggest that Uev1A promotes cell survival under serum starvation stress through the AKT-FOXO1-BIM axis in breast cancer cells, and identified a potential therapeutic target in the treatment of both triple negative and estrogen receptor positive breast cancers.

## Supplementary information

**Additional file 1: Figure S1**. Effects of *UEV1C* and *MMS2* overexpression on the ATK pathway in breast cancer cells. **Figure S2**. Colony formation assay under serum-deprived conditions. **Figure S3**. Growth curve of Uev1A depleted breast cancer cells under serum-supplemented conditions. **Figure S4**. *UEV1A* transcript levels in shUEV1 restored UEV1A breast cell lines. **Figure S5**. Uev1A promotes cell survival under serum starvation conditions through the AKT pathway in breast cancer cells. **Figure S6**. Inhibition of NF-κB pathway by Bay11-7082 treatment. **Figure S7**. Effects of Uev1 depletion on chemoresistance of breast cancer cells. **Figure S8**. Uev1A inhibits apoptosis through the AKT pathway in breast cancer cells.

## Data Availability

The datasets used and/or analyzed during the current study are available from the corresponding author upon reasonable request.
