# Supplementary material for: Uev1A promotes breast cancer cell survival and chemoresistance through the AKT-FOXO1-BIM pathway
Source: Cancer Cell Int. 2019 Dec 9;19:331. doi: 10.1186/s12935-019-1050-4 (PMC6902549; doi:10.1186/s12935-019-1050-4)
Supplement: Supplementary file 1 — Additional file 1: Figure S1. Effects of UEV1C and MMS2 overexpression on the ATK pathway in breast cancer cells. Figure S2. Colony formation assay under serum-deprived conditions. Figure S3. Growth curve of Uev1A depleted breast cancer cells under serum-supplemented conditions. Figure S4. UEV1A transcript levels in shUEV1 restored UEV1A breast cell lines. Figure S5. Uev1A promotes cell survival under serum starvation conditions through the AKT pathway in breast cancer cells. Figure S6. Inhibition of NF-κB pathway by Bay11-7082 treatment. Figure S7. Effects of Uev1 depletion on chemoresistance of breast cancer cells. Figure S8. Uev1A inhibits apoptosis through the AKT pathway in breast cancer cells. [file 12935_2019_1050_MOESM1_ESM.docx]

**Additional figure legends**

**Figure S1. Effects of *UEV1C* and *MMS2* overexpression on the ATK pathway in breast cancer cells.** AKT and its phosphorylation were monitored by western blot in pcDNA4.0/TO/HA(+) vector (CK), *UEV1C* or *MMS2* stably transfected (**A**) MDA-MB-231-TR cells treated with doxycycline or (**B**) MCF7 cells. The expression levels of ectopic Uev1C and Mms2 were monitored by an anti-HA antibody.

**Figure S2. Colony formation assay** **under serum-deprived conditions.** 1x10^5^ cells were seeded in 6-well culture plates. The cells were cultured for 7 days with serum-free medium. Colonies were fixed with methonol, stained with trypan blue (0.4% w/v) and counted using a microscope. Each sample was measured in triplicate and repeated at least 2 times. * *P*<0.05.

**Figure S3. Growth curve of Uev1A depleted breast cancer cells under serum-supplemented conditions. (A)** Growth curve of non-specific target (shCK) and shUEV1 MDA-MB-231 cell lines (shUEV1-1 and shUEV1-2) under serum-supplemented conditions. Then cells were harvested by trypsinization at different time point and stained with trypan blue. Viable cells were counted using a hematocytometer and an inverted microscope. **(B)** Growth curve of non-specific target (shCK) and shUEV1 MCF7 cell line (shUEV1-8) under serum-supplemented conditions. Experimental conditions were as described in Fig. S3A. Each sample was measured in triplicate and repeated 2 times. * *P*<0.05.

**Figure S4. *UEV1A* transcript levels in shUEV1 restored UEV1A breast cell lines. (A)** Two shUEV1 MDA-MB-231 cell lines were transfected with the pcDNA4.0/TO/HA(+) vector expressing *UEV1A* (shUEV1-1+UEV1A and shUEV1-2+UEV1A). Non-specific shRNA targeted MDA-MB-231 cells were also transfected with the pcDNA4.0/TO/HA(+) vector to serve as a control. *UEV1A* transcript levels in shCK and shUEV1 lines were determined by qRT-PCR. **(B)** The MCF7 shUEV1-8 cell line was transfected with pcDNA4.0/TO/HA(+) vector expressing *UEV1A* (shUEV1-8+UEV1A). The non-specific shRNA targeted MCF7 cells were transfected with the vector alone (shCK+CK). The *UEV1A* transcript levels in shCK and shUEV1 line were determined by qRT-PCR. ** *P*<0.01.

**Figure S5. Uev1A promotes cell survival under serum starvation conditions through the AKT pathway in breast cancer cells. (A)** *UEV1A* overexpressed MDA-MB-231-TR cells were treated with Perifosine. After 24 hrs, the AKT pathway proteins were examined by western blot in the whole-cell extract (WCE) or nuclear fraction (N) in *UEV1A* overexpressed cells alone (UEV1A) or treated with 5 μM Perifosine (1A+Pe), or vector only (CK). The expression levels of ectopic Uev1A were monitored by an anti-HA antibody. **(B,C)** Growth curve of control (CK) and *UEV1A*-overexpressed MDA-MB-231-TR **(B)** and MCF7 **(C)** cells under serum-deprived conditions. Experimental conditions were as described in Fig. 2C except that cells were treated with 5 μM Perifosine (Pe+). Each sample was measured in triplicate and repeated at least 2 times. * *P*<0.05; ** *P*<0.01

**Figure S6. Inhibition of NF-κB pathway by Bay11-7082 treatment.** Cells were treated with or without 40 μM Bay11-7082 for 4 hours. Then whole-cell extract (WCE) or nuclear fraction (N) were prepared for western blotting analysis using an anti-p65 antibody to assess the protein level of p65 in whole-cell extract or nuclear fraction.

**Figure S7. Effects of Uev1 depletion on chemoresistance of breast cancer cells**. **(A,B)** Non-specific (shCK) and shUEV1 MDA-MB-231 cells were seeded onto 6-well culture plates. After a 4-hr exposure to various doses of Paclitaxel **(A)** or Doxorubicin **(B)**, the cells were cultured for an additional 7 days with drug-free medium containing 10% FBS**.** Cell viability was determined and presented as described in Figure 6. **(C,D)** Non-specific target (shCK) and shUEV1 MCF7 cells were seeded onto 6-well culture plates. After a 4-hr exposure to Paclitaxel **(C)** or Doxorubicin **(D)**, the cells were cultured for an additional 7 days with drug-free medium containing 10% FBS. Cell viability assay was as described in Figure 6. * *P*<0.05; ** *P*<0.01.

**Figure S8. Uev1A inhibits apoptosis through the AKT pathway in breast cancer cells.**

MDA-MB-231 (**A,B**) or MCF7 (**C,D**) cells were per-treated with 5 µM Perifosine (Pe) for 12 hours and then exposed to Paclitaxel (**A,C**) or Doxorubicin (**B,D**), harvested at different time points and the protein levels of total PARP and cleaved-PARP were detected by western blot. The ectopic Uev1A was monitored by an anti-HA antibody.


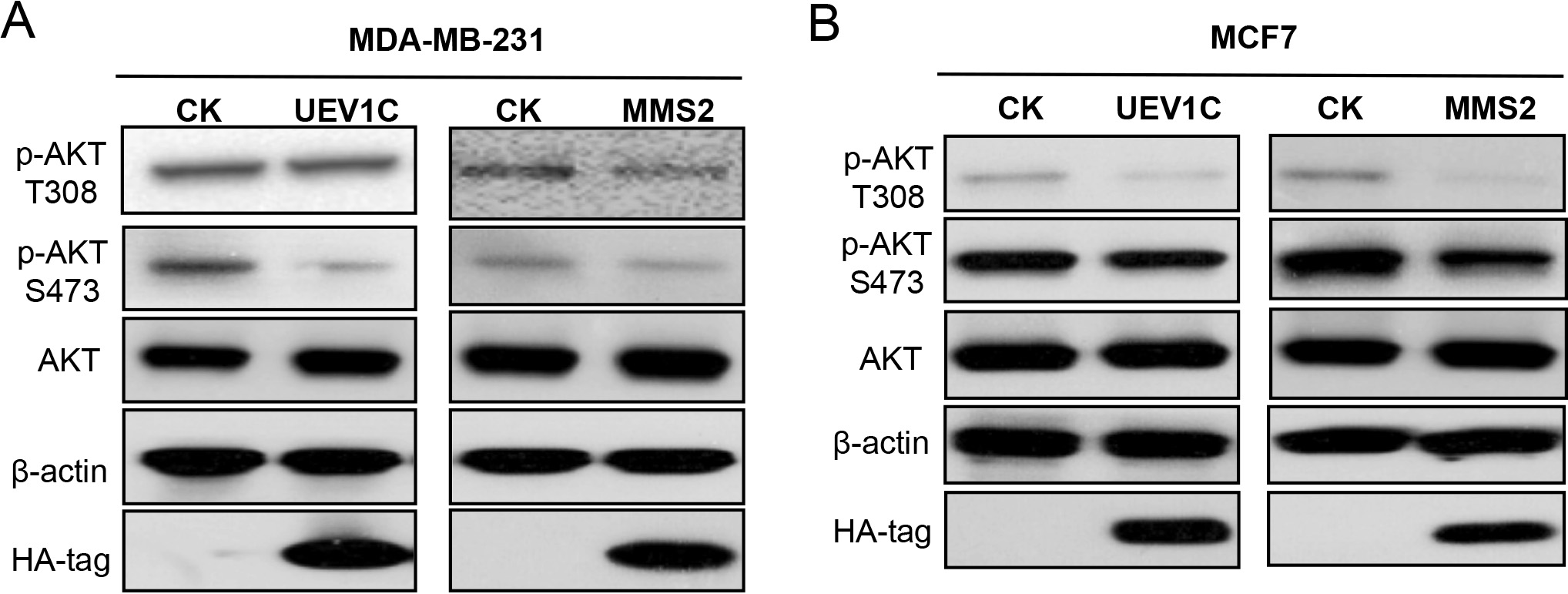


Figure S1


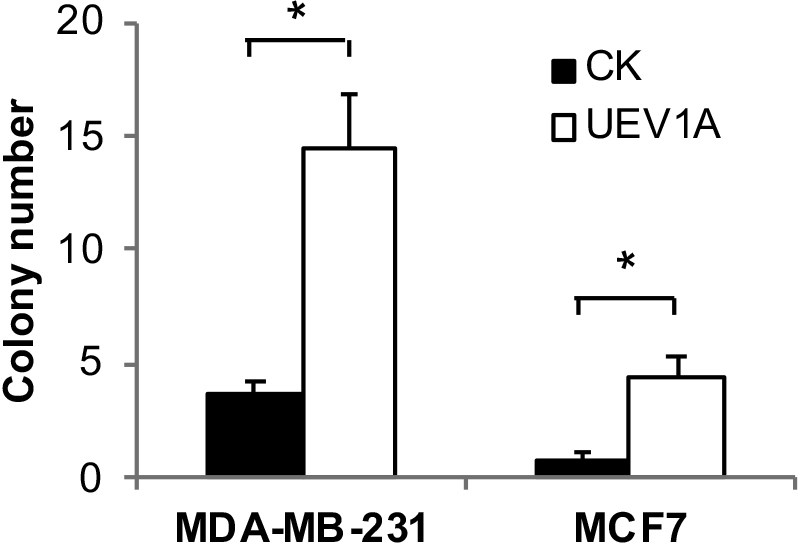


Figure S2


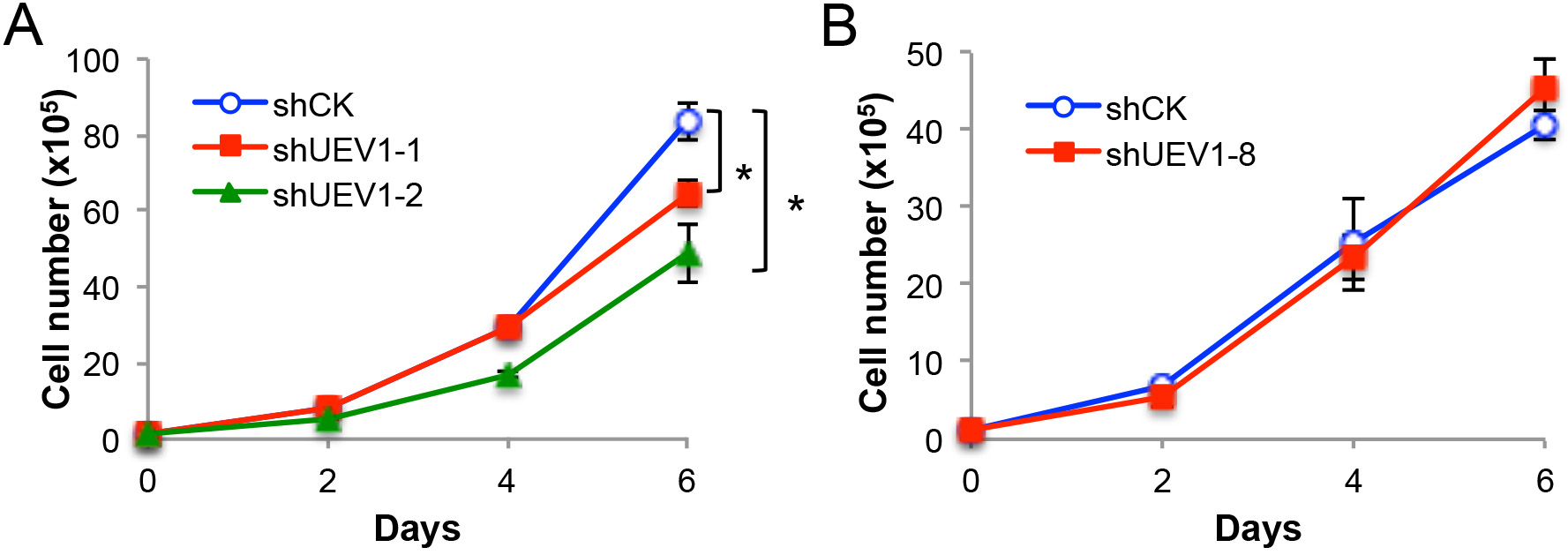


Figure S3


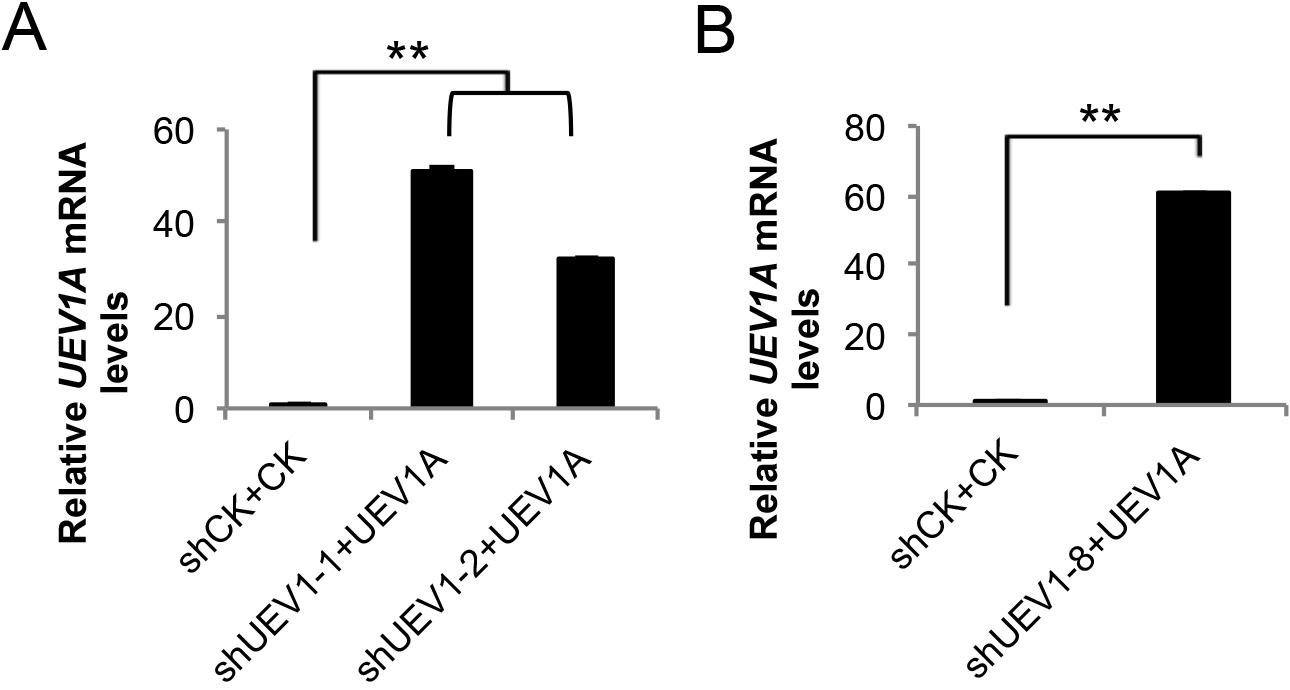


Figure S4


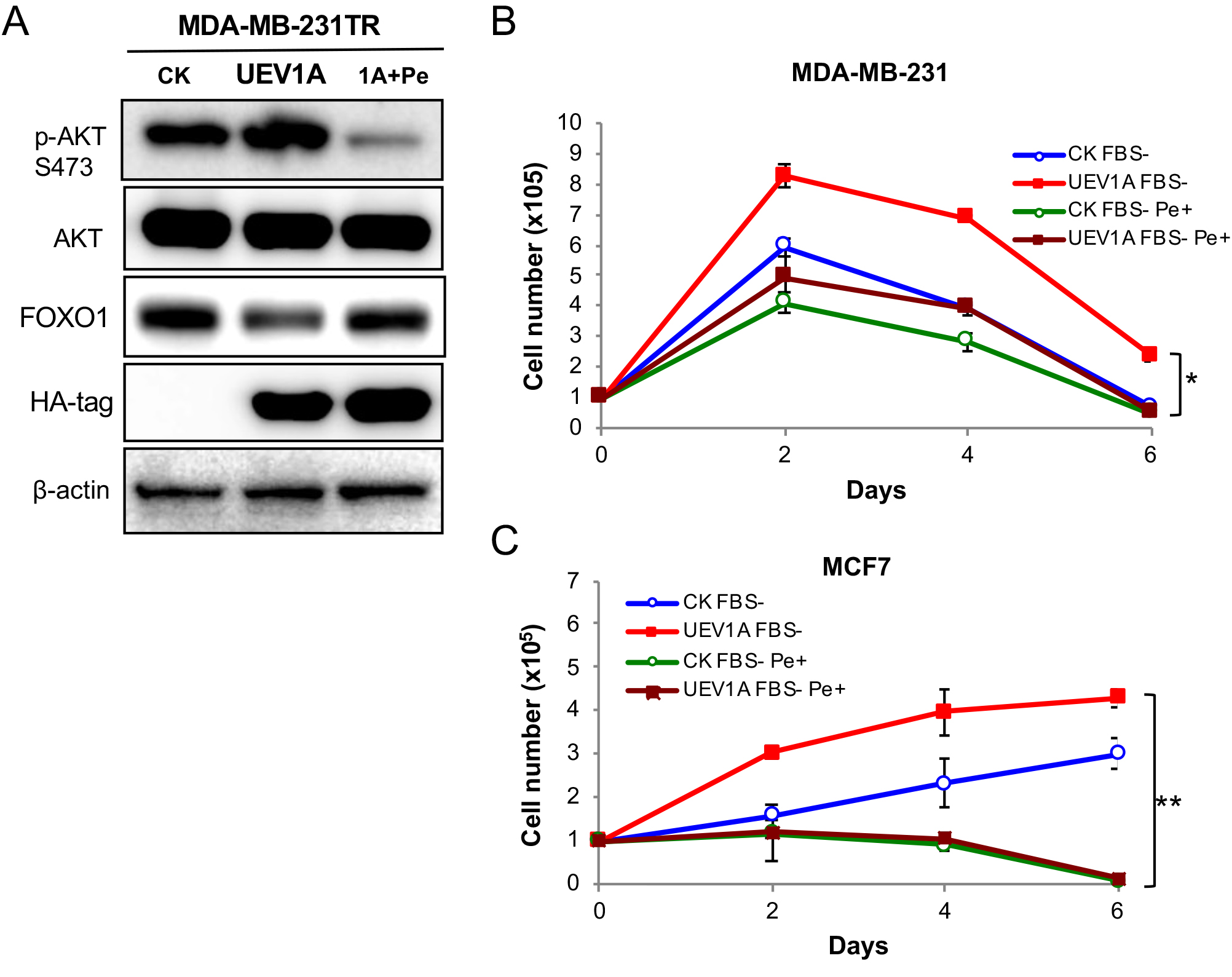


Figure S5


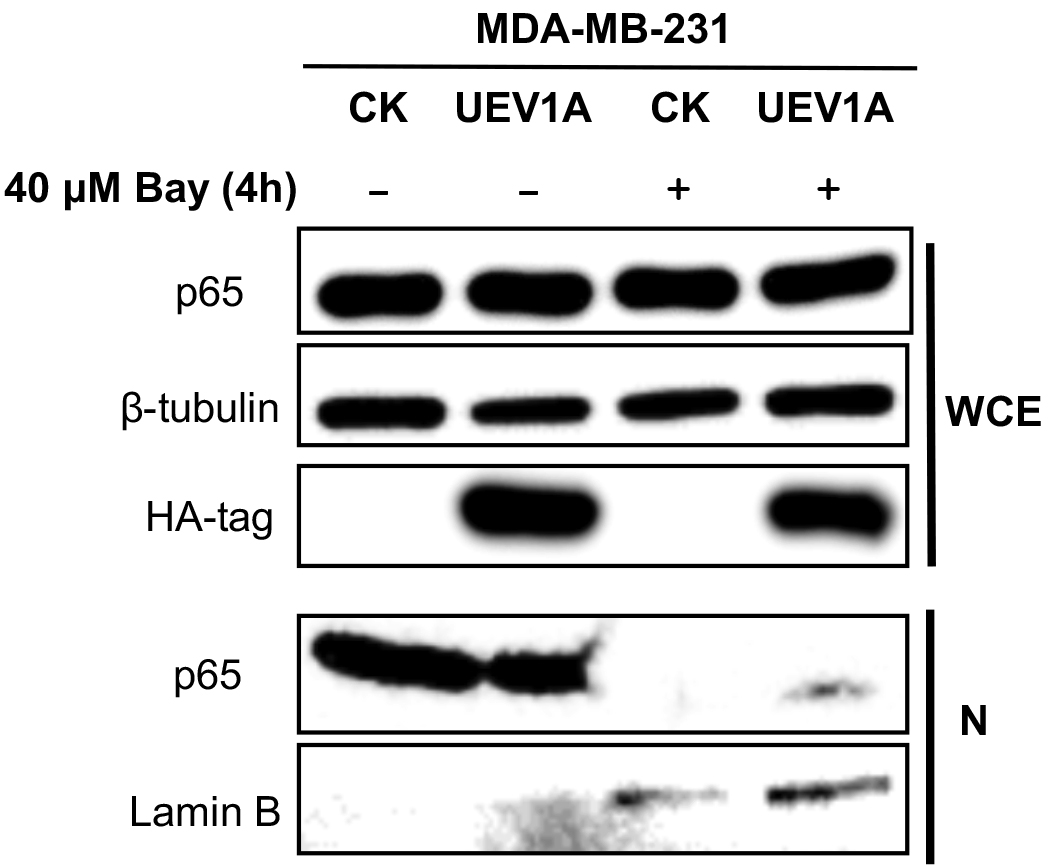


Figure S6


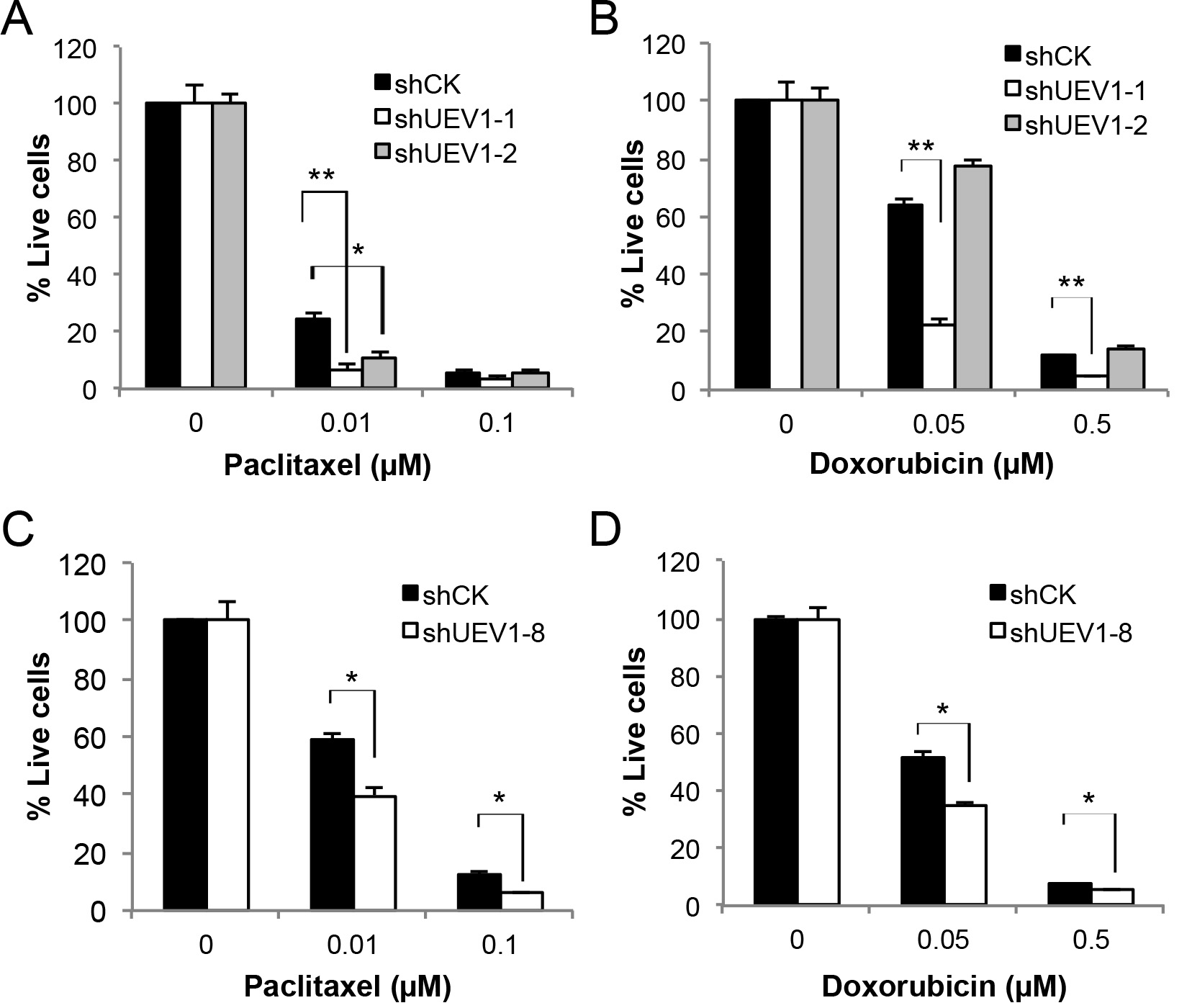


Figure S7


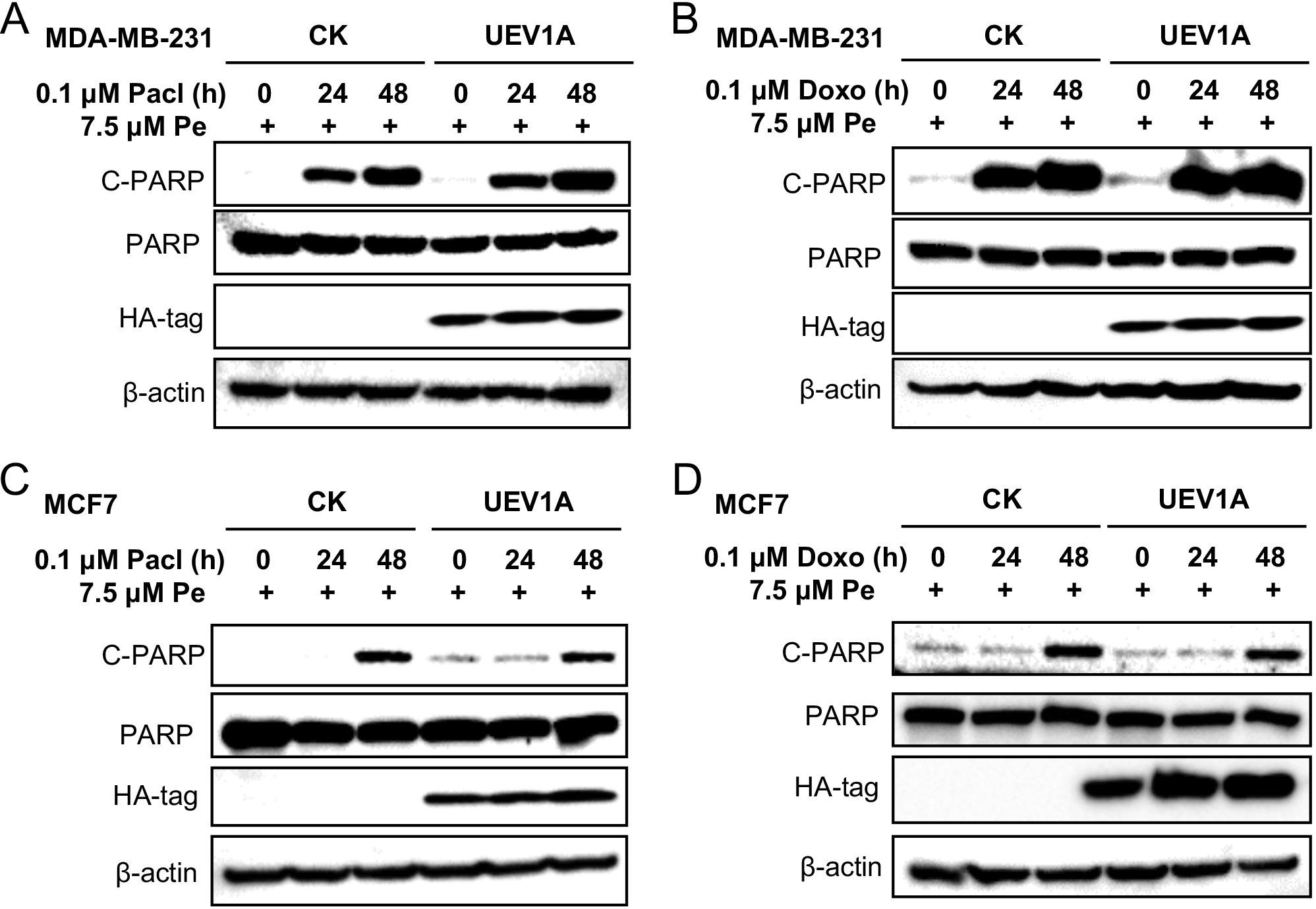


Figure S8
